# A descriptive study on health workforce performance after decentralisation of health services in Uganda

**DOI:** 10.1186/1478-4491-10-41

**Published:** 2012-11-07

**Authors:** George William Lutwama, Janetta Hendrika Roos, Bethabile Lovely Dolamo

**Affiliations:** 1 Graduate, Department of Health Studies, Unisa & IMA World Health Sudd Health Project, IMA World Health, 500 Main Street, PO Box 429, New Windsor, MD, 21776, USA; 2 Department of Health Studies, PO Box 392, Pretoria, Unisa 0003, South Africa

**Keywords:** Health workers, Health workforce, Decentralisation, Performance, Health sector reforms

## Abstract

**Background:**

Uganda, like many developing countries, is committed to achieving the Millennium Development Goals (MDGs) by 2015. However, serious challenges prove to hamper the attainment of these goals, particularly the health related MDGs. A major challenge relates to the human resources for health. The health system in Uganda was decentralised in the 1990s. Despite the health sector reforms, the services have remained significantly deficient and performance of health workers is thought to be one of the contributing factors. The purpose of this study was, therefore, to investigate the performance of health workers after decentralisation of the health services in Uganda in order to identify and suggest possible areas for improvement.

**Methods:**

A cross-sectional descriptive survey, using quantitative research methods was utilised. A structured self-administered questionnaire was used to collect quantitative data from 276 health workers in the districts of Kumi, Mbale, Sironko and Tororo in Eastern Uganda. The health workers included doctors, clinical officers, professional nurses and midwives. The sample was selected using stratified random sampling. The data was analysed using SPSS version 18.0 and included both univariate and bivariate analysis. The results were presented in tabular and text forms.

**Results:**

The study revealed that even though the health workers are generally responsive to the needs of their clients, the services they provide are often not timely. The health workers take initiatives to ensure that they are available for work, although low staffing levels undermine these efforts. While the study shows that the health workers are productive, over half (50.4%) of them reported that their organisations do not have indicators to measure their individual performance. The findings indicate that the health workers are skilled and competent to perform their duties. In general, the results show that health workers are proficient, adaptive, proactive and client-oriented.

**Conclusion:**

Although Uganda is faced with a number of challenges as regards human resources for health, the findings show that the health workers that are currently working in the health facilities are enthusiastic to perform. This may serve as a motivator for the health workers to improve their performance and that of the health sector.

## Background

One of the recent priorities meant to improve effectiveness of the health systems in Uganda is investment in human resources for health. The World Health Organization declared 2006 to 2015 a decade for the health workforce with high emphasis on the performance of human resources for health [1]. The World Health Organization (WHO) defines the performance of health workers in terms of four dimensions that include responsiveness, productivity, competence and availability [1]. Hence, a performing health workforce is one that operates in ways that are reasonable, responsive and efficient to achieve favourable health outcomes [1]. The First Global Forum on Human Resources for Health held in 2008 in Kampala, issued a declaration that emphasises the need for equitable distribution as well as retention of skilled, productive and responsive health workers [2].

The public health system in Uganda has been decentralised to the districts since the health sectors reforms of the early 1990s [3-5]. The public health care system comprises the national hospital, regional referral hospitals and the district health care systems. The district health system comprises the district hospitals, health centres levels II, III, and IV and the village health teams (VHTs). The district hospital is the highest level of health care in the district. The district health care system is further subdivided into health sub-districts or a level IV health centre, which is aligned to a county or parliamentary constituency. Each county is sub divided into sub-counties. The health care services at this level are provided by a level III health centre. The sub-counties are further sub-divided into parishes or wards. The health care facility at this level is a level II health centre. This is the lowest level of health care facility that offers primary health care services. Lastly, parishes or wards are divided into villages. At this level there are VHTs, which are teams of local residents appointed to oversee the performance of health activities in their communities [6,7].

Despite the health sector reforms, the health services in Uganda have remained poor. The performance of health workers together with lack of adequate support from the central and local governments are believed to play a part in the stagnation and decline of some of the priority health indicators under the current Health Sector Strategic Plan (HSSP III) [7]. For instance, the proportion of children under one year who have received three doses of the pentavalent vaccine as per schedule reduced from 89% in the financial year 2005 to 2006 to 78% in 2007 to 2008 against the target of 87%. The tuberculosis (TB) cure rate reduced from 73% to 68.4% during the same period. The proportion of sick children under age five years, who were seen by a health worker using the Integrated Management of Childhood Illnesses (IMCI) guidelines, reduced from 45% at baseline to 30% (2007 to 2008), below the target of 55%. The outpatient new attendance rate stagnated at 80% for the past consecutive two years [8].

The Uganda press reports provide significant evidence regarding health worker performance problems in the districts, such as absenteeism, neglect of patients, drug pilferage and poor staff motivation. Evidence shows that the Minister of Health in Uganda once blamed decentralisation as the cause for poor delivery of health service in the country [9]. As a result of the deterioration of the health services, the President of Uganda wrote a letter to the Minister of Health in April 2008 demanding an explanation for health sector failures [10]. Whatever the case, the inadequate performance of individual health workers leads to poor health sector performance. It was, therefore, necessary to investigate the performance of the health workforce after decentralisation to identify the root cause of the problems.

### Objective

The objective of this study was to investigate the performance of health workers after decentralisation of health services in Uganda in order to identify areas for improvement. Specifically, the study aimed at examining the performance based on the WHO dimensions of availability, productivity, competence and responsiveness. The study also aimed at identifying the behaviours of health workers that facilitate or hinder their performance.

## Methods

The study was a cross-sectional and descriptive survey using quantitative research methods. The study population comprised these cadres of health workers: doctors, clinical officers and professional nurses working in health facilities in the Kumi, Mbale, Sironko and Tororo districts. To ensure representativeness and avoid bias, stratified random sampling was used [11,12]. Each district constituted a stratum. Within each district the sample was further stratified by the profession and type of health facility where they work. Using the lists of health workers obtained from the district health officers of the respective districts, a sample was randomly drawn from each stratum. A total of 331 health workers representing approximately 40% of the target population were included in the final sample. Of these, 83.4% (n = 276) completed and returned the questionnaires.

A structured self-administered questionnaire was used to collect data concerning the dimensions of performance as well as the individual behaviours for performance. The questionnaire was based on a five-point Likert scale (where 1 = strongly disagree; 2 = disagree; 3 = undecided; 4 = agree and 5 = strongly agree). Four questions elicited information about individual behaviours for performance with a number of sub-items as follows; proficiency behaviour (five items); adaptive behaviours (four items); proactive behaviours (four items) and client orientation behaviours (ten items). There were also four questions about the dimensions of performance as follows: availability (ten items); responsiveness (nine items); productivity (six items) and competence (nine items). The questionnaires were in English, which is the official working language in Uganda.

The pre-testing of the questionnaire was done at two levels. The first level involved seven experts in the field of study and the second level was with twelve experienced health workers (seven professional nurses and midwives, three clinical officers, and two medical doctors). These health workers were selected using convenience sampling from the hospitals of Kumi and Mbale. Results from the pre-test assisted with minor modifications in the wording of some of the questionnaire items. Following the pre-test and modifications, the first author (GWL) together with the trained research assistants delivered the questionnaires, a consent form and a letter explaining the reasons for conducting the study to the respondents. This was implemented from April 2010 to June 2010. The questionnaires were left with the respondents and collected within two weeks to allow them ample time to complete. Overall, the time for completing the questionnaire ranged from 30 to 45 minutes.

### Data analysis

The returned self-administered questionnaires were cross-checked for completeness, coded and entered into a computer using the Statistical Package for Social Sciences (SPSS) version 18.0. The data were cleaned and analysed using the same version. For the purposes of the data presentation and interpretation, the categories ‘strongly disagree’ and ‘disagree’ were combined together to represent ‘disagree’. Similarly, ‘strongly agree’ and ‘agree’ were combined to represent ‘agree’. The results were presented in tabular form. Statistical analysis was done using both univariate and bivariate statistics. Bivariate analysis using Spearman rho correlation was used to determine association between two variables. Spearman rho correlation (*rs*) was used mainly where the data were based on the ordinal scale and, were therefore, not normally distributed [13]. The internal consistency of the scales was assessed using Cronbach’s alpha (*α*) and ranged from 0.76 to 0.91. The average alpha (*α*) was found to be 0.84, which was adequate [14].

External validity was ascertained by way of stratified random sampling, and obtaining a correct composition and adequate sample size. Furthermore, the researcher collected data from all levels of health facilities including hospitals and health centres. Internal validity was ensured by constructing questionnaire items based on the study objective and extensive literature review, as well as voluntary participation of the health workers.

### Ethical considerations

Ethical clearance was obtained from the Research and Ethics Committee at the Department of Health Studies of the University of South Africa; the Uganda National Council for Science and Technology (UNCST); and the Institutional Review Board of Mbale regional hospital. In the field, permission to collect data was obtained from the district officials, hospital medical superintendents and heads of the health sub-districts. Informed written consent was obtained from all respondents and their privacy was respected at all levels. The respondents were informed of their freedom to withdraw from the study at any time or not to answer any specific questions. Confidentiality was maintained by protecting data collected from unauthorised access and the respondents remained anonymous.

## Results and discussion

### Characteristics of the respondents

The majority of respondents (63.8%) were female and aged between 30 and 49 years (62.4%); 74.6% were either professional nurses or midwives, 18.5% were clinical officers and 6.9% were medical doctors. Most respondents (53.6%) held diplomas. The majority of respondents (73.6%) had more than five years of professional experience. Of the respondents, 56.9% were working in health centres (levels II, III, IV) while 43.1% were hospital based. As many as 59.8% health workers were working in rural areas. With regard to district of work, 38.4% were from Mbale, 23.6% from Tororo, 19.9% from Kumi and 18.1% from Sironko (see Table 1).

**Table 1:** Characteristics of the respondents (n = 276)

**Demographic characteristics**	**Number**	**Percentage (%)**
**Gender**		
Female	176	63.8
Male	100	36.2
**Age, years**		
20 to 29	50	18.1
30 to 39	115	41.7
40 to 49	57	20.7
50 to 59	54	19.6
60 and above	0	0.0
**Current profession**		
Medical doctor	19	6.9
Clinical officer	51	18.5
Registered nurse/midwife	111	40.2
Enrolled nurse/midwife	95	34.4
**Highest qualification**		
Doctoral degree	2	0.7
Master’s degree	4	1.4
Postgraduate certificate/diploma	5	1.8
Bachelor’s degree	23	8.3
Diploma	148	53.6
Certificate	94	34.1
**Years of experience in health profession**		
0 to 5	73	26.4
6 to 10	68	24.6
11 to 15	43	15.6
16 to 20	26	9.4
21 and above	66	23.9
**Type of health facility**		
Hospital	119	43.1
Health centre IV	56	20.3
Health centre III	80	29.0
Health centre II	21	7.6
**District of work**		
Kumi	55	19.9
Mbale	106	38.4
Sironko	50	18.1
Tororo	65	23.6
**Location of health facility**		
Urban	111	40.2
Rural	165	59.8

### Dimensions for measurement of performance of health workers

The performance of health workers was measured using four dimensions namely responsiveness, availability, productivity and competence.

#### Responsiveness of health workers

Table 2 shows that all respondents (100.0%) know what is expected of them. Most health workers (89.9%) agreed that their clients are always satisfied with their services. The majority of the respondents (79.7%) indicated that they get professional support to improve their performance. A total of 70.3% health workers indicated that they clearly know who they serve and many of them (69.9%) are always willing to address the clinical and emotional demands of their clients. As many as 59.4% health workers agreed that the stakeholders are satisfied with the health workers’ cooperation and the quality of service they provide. However, as many as 40.6% health workers indicated that clients were not satisfied with the quality of service they receive and 30.4% of respondents indicated that the clients were not satisfied with the level of cooperation they get from the health workers. Half of the respondents (50%) indicated that rarely do the stakeholders complain about individual health workers, 30.1% were undecided and 19.9% disagreed. It is, however, noted that 60.1% of the respondents disagreed that the patients were satisfied with the timeliness of the services, while 10.1% were uncertain.

**Table 2:** Items concerning the responsiveness of health workers (n = 276)

**Item description**	**Disagree**		**Undecided**		**Agree**		**Total**	
	**n**	**%**	**n**	**%**	**n**	**%**	**n**	**%**
Clients are always satisfied with the friendly services offered by health workers	28	10.1	0	0.0	248	89.9	276	100.0
Clients are satisfied with the quality of services we provide	112	40.6	0	0.0	164	59.4	276	100.0
Clients are satisfied with the timeliness of the services	166	60.1	28	10.1	82	29.7	276	99.9
Complaints from stakeholders towards individual health workers are rare	55	19.9	83	30.1	138	50.0	276	100.0
Stakeholders are satisfied with the health workers’ cooperation	84	30.4	28	10.1	164	59.4	276	99.9
Health workers clearly know who they serve	82	29.7	0	0.0	194	70.3	276	100.0
Health workers are always willing to address the clinical and emotional demands of the patients	55	19.9	28	10.1	193	69.9	276	99.9
Health workers get professional support to improve their performance	56	20.3	0	0.0	220	79.7	276	100.0
When at work I know what is expected of me	0	0.0	0	0.0	276	100.0	276	100.0

Generally, from their own point of view, the health workers demonstrated satisfactory levels of service and responsiveness towards their clients. However, from their own point of view, the services that health workers provide are not timely. Health workers must demonstrate the ability and willingness to understand and address both the clinical and emotional needs of their clients [15,16]. This study revealed that health workers were not providing timely services. This can be attributed to organisational constraints such as the low staffing levels, heavy workload, poor work flow structures and probably lack of some essential drugs and equipment necessary for performance [17]. It is essential that health workers are responsive to the demands of the communities they serve. This allows for a more holistic approach to health service provision, taking into consideration both the technical aspects and client satisfaction, thereby guaranteeing quality [18].

#### Availability of health workers

Most of the health workers (90.2%) indicated that their health facilities have attendance registers that are completed by all staff. Similarly, 90.2% of health workers agreed that they put in much effort when they are on duty. Approximately ninety percent (89.9%) of the health workers indicated that they are always available when needed. A total of 60.1% indicated that they are always present at work during the official working hours and also agreed that their organisations have clear retention policies. As many as 59.8% agreed that the staff attendance in their organisations was good, however, 40.2% disagreed. There were mixed views in the responses from the health workers concerning patients’ waiting-times. A total of 40.6% of health workers agreed that waiting-times were short, while 39.5% disagreed and 19.9% were uncertain (see Table 3).

**Table 3:** Items concerning the availability of health workers (n = 276)

**Item description**	**Disagree**		**Undecided**		**Agree**		**Total**	
	**n**	**%**	**n**	**%**	**n**	**%**	**n**	**%**
This organisation has a retention policy with clear strategies	55	19.9	55	19.9	166	60.1	276	99.9
I am always available when my services are required	28	10.1	0	0.0	248	89.9	276	100.0
This facility has adequate numbers of health workers to deliver the services	138	50.0	28	10.1	110	39.9	276	100.0
The rural facilities are as well staffed as the urban ones	221	80.1	27	9.8	28	10.1	276	100.0
This facility has an attendance register which is filled by every staff member	27	9.8	0	0.0	249	90.2	276	100.0
I am always present during the official working hours	82	29.7	28	10.1	166	60.1	276	100.0
I put in much effort when I am on duty	27	9.8	0	0.0	249	90.2	276	100.0
Staff attendance in this organisation is good	111	40.2	0	0.0	165	59.8	276	100.0
The workload in this facility is manageable	111	40.2	56	20.3	109	39.5	276	100.0
In this facility the patient’s waiting-time is short	109	39.5	55	19.9	112	40.6	276	100.0

A large proportion of health workers (80.1%) disagreed that the rural facilities are as well staffed as the urban ones. Fifty percent of the health workers disagreed that their health facilities have adequate numbers of staff to deliver the services, 39.9% agreed and 10.1% were undecided. There were also mixed views among health workers concerning the workload. As many as 40.2% health workers indicated that the workload at their health facilities is not manageable while 39.5% stated that the workload was manageable and 20.3% were undecided (see Table 3).

The results of this study demonstrate that health workers try their best to be present at work. The reported high rate of disagreement among health workers regarding adequacy of staffing (Table 3) may probably explain high workload and long patients’ waiting-times [17]. It is however, important to note that availability of health workers on its own cannot directly improve the health outcomes unless there is effective drug supply, functioning facilities and good clinical practice [19]. A well performing health workforce is a significant component of a strong health system that together with other social determinants can improve the health status of the population. As the numbers of health workers drop, the ability of systems to deliver health services also reduces [1,20]. Hence, the maldistribution of health workers between rural and urban health facilities as reported by the health workers in this study (Table 3), could affect the quality of health care and waiting-times may be longer [21].

#### Productivity of health workers

Of the health workers, 80.1% agreed that their skills are suited for the type of work they do. The majority of health workers (79.7%) indicated that availability of drugs and equipment improves their productivity. Most health workers (70.3%) indicated that their health facility management encourages them to perform. As many as 60.5% of workers agreed that they spend most of their time at work attending to the patients and that their productivity is measured according to the number of clients they attend (see Table 4).

**Table 4:** Items concerning the productivity of health workers (n = 276)

**Item description**	**Disagree**		**Undecided**		**Agree**		**Total**	
	**n**	**%**	**n**	**%**	**n**	**%**	**n**	**%**
My productivity is measured according to the number of patients I attend to	109	39.5	0	0.0	167	60.5	276	100.0
This organisation has indicators for measuring staff productivity	139	50.4	27	9.8	110	39.9	276	100.1
I spend most of my time attending to patients	81	29.3	28	10.1	167	60.5	276	99.9
My skills are suited for the type of work I do	28	10.1	27	9.8	221	80.1	276	100.0
My productivity is increased by availability of drugs and equipment	0	0.0	56	20.3	220	79.7	276	100.0
The management structures in this facility encourage the performance of health workers	54	19.6	28	10.1	194	70.3	276	100.0

A total of 39.5% of the health workers disagreed that their productivity is measured according to the number of clients they manage and 29.3% disagreed that they spend most to their time attending to clients, while 10.1% were undecided. With regard to indicators for measuring performance, 50.4% of respondents indicated that their organisations do not have indicators for measuring productivity of health workers, 39.9% agreed, and 9.8 % were uncertain (see Table 4).

Generally, the health workers’ self-reporting indicates that they possess adequate skills necessary to perform their jobs. The workers stressed the importance of having adequate supplies of drugs and equipment to support their productivity. The results, however, show that the measurement of productivity of health workers is generally lacking in most health facilities. Much as the health workers have adequate skills, their productivity can be limited by a host of factors like lack of equipment, supplies or drugs, poor management structures and low salaries [22,23]. This study revealed that some health workers (39.5%) did not seem to understand how their productivity was measured and slightly over half did not know that there were indicators for measuring productivity. There is, therefore, a need to develop and communicate clear indicators for measuring productivity.

#### Competence of health workers

All the health workers (100.0%) indicated that they have mastered the skills necessary to do their work and are confident about their prescribing practices, and all said that they improve their knowledge and skills through continuous professional education, and use their knowledge and skills to improve the safety of their clients. All health workers (100.0%) agreed that their attitude towards patient care is good and always make timely referrals of patients in need of further treatment and care (see Table 5).

**Table 5:** Items concerning the competence of health workers (n = 276)

**Item description**	**Disagree**		**Undecided**		**Agree**		**Total**	
	**n**	**%**	**n**	**%**	**n**	**%**	**n**	**%**
I am confident my ability to do my job	28	10.1	0	0.0	248	89.9	276	100.0
I have mastered the skills necessary to perform my job	0	0.0	0	0.0	276	100.0	276	100.0
I am confident about my prescribing practices	0	0.0	0	0.0	276	100.0	276	100.0
I always improve my knowledge and skills through continuous professional education	0	0.0	0	0.0	276	100.0	276	100.0
My attitude toward the care of patients is good	0	0.0	0	0.0	276	100.0	276	100.0
I use my knowledge and skills to improve safety of patients	0	0.0	0	0.0	276	100.0	276	100.0
I always make timely referral of patients that are in need of specialised treatment	0	0.0	0	0.0	276	100.0	276	100.0
I have good communication skills	0	0.0	0	0.0	276	100.0	276	100.0
I am able to use the available communication technology to support patient care	0	0.0	0	0.0	276	100.0	276	100.0

All the health workers (100.0%) indicated that they have good communication skills and are able to use the available technology to support patient care. The majority of health workers (89.9%) agreed that they are confident about their ability to perform their jobs.

Generally, the results show that the health workers are competent and this is highly likely to enhance their performance. High level of knowledge, skills and abilities among health workers are necessary for effective performance [24]. Health workers also reported that they have good communication skills. This is very important because listening, guidance, clear expectations and feedback are prerequisites for better performance [25]. The health workers in this study reported that they make timely referral when required. This is consistent with findings from a study done in India, where maternal health outcomes improved as a result of good skills in identifying complicated obstetric cases, good counselling and communication skills [26]. The findings of this study however, are contrary to what is reported in Niger, where rural health workers lack the competence to communicate, counsel and convince patients with complications requiring referral to higher level facilities [27].

### Behaviour of health workers for performance

#### Proficiency behaviours

All the health workers (100.0%) agreed that they communicate openly with each other, coordinate their work amongst themselves and support their co-workers when requested. Most health workers (80.1%) indicated that they use standard procedures to ensure that their core tasks are properly done while 19.9% disagreed. Furthermore, 79.9% of the health workers indicated that they portray a positive image of their organisations and 10.1% were either in disagreement or uncertain (see Table 6).

**Table 6:** Items concerning the individual proficiency behaviours of health workers (n = 276)

**Item description**	**Disagree**		**Undecided**		**Agree**		**Total**	
	**n**	**%**	**n**	**%**	**n**	**%**	**n**	**%**
I always use standard procedures to ensure that my core tasks are completed properly	55	19.9	0	0.0	221	50.4	276	100.0
I coordinate my work with my co-workers	0	0.0	0	0.0	276	50.7	276	100.0
I communicate openly with my co-workers	0	0.0	0	0.0	276	100.0	276	100.0
I provide help to my co-workers when asked	0	0.0	0	0.0	276	100.0	276	100.0
I portray a positive image of my organisation to others such as the patients	28	10.1	28	10.1	220	79.7	276	99.9

Overall, health workers display satisfactory proficiency behaviours that are likely to support good performance. This finding contradicts what was found by a Kenyan study [17], which established that health workers working in hospital paediatric departments were not cooperating and communicating openly with each other and this was affecting their performance and the quality of care given to patients. Some researchers have established that behaviours that involve cooperation, creativity and self-development contribute to job performance [28]. Additionally, role clarity is known to be one of the strongest predictors of individual, team, and task organisational proficiency behaviours for performance [29].

#### Adaptive behaviours

All health workers (100.0%) agreed that they adjust well to changes in their core tasks and have learned new skills to help them adjust to change. Most health workers (89.9%) indicated that they are flexible with regard to the overall changes within their organisations and 10.1% disagreed. Similarly, 80.1% of the health workers agreed that they respond constructively to changes in the way their teams work (see Table 7).

**Table 7:** Items concerning the adaptive behaviours of health workers (n = 276)

**Item description**	**Disagree**		**Undecided**		**Agree**		**Total**	
	**n**	**%**	**n**	**%**	**n**	**%**	**n**	**%**
I adjust well to changes in my core tasks	0	0.0	0	0.0	276	100.0	276	100.0
I have learned new skills to help me adjust to changes in my core tasks	0	0.0	0	0.0	276	100.0	276	100.0
I respond constructively to changes in the way my team works	55	19.9	0	0.0	221	80.1	276	100.0
I am flexible with regard to the overall changes in my organisation	28	10.1	0	0.0	248	89.9	276	100.0

These results indicate that the health workers display positive individual adaptive behaviours, which can contribute to good performance. This is contrary to what was found in Kenya where health workers were reported to have inadequacies in adapting or responding to new clinical practices or guidelines due to lack of perceived benefits linked to their uptake [17]. In that study, senior and older health workers were often reported to be stuck in the patterns of previous clinical practice although there were some exceptions [17]. This problem was attributed to the lack of experience in being compelled to change by emerging knowledge. Openness to change is known to be a strong predictor for adaptive behaviour. This can enhance task performance for an individual, teams and the organisation as a whole [29].

#### Proactive behaviours

From Table 8, it is clear that all health workers (100.0%) indicated that that they have initiated better ways of performing their core tasks. A large proportion of health workers (90.2%) agreed that they have developed new and improved methods to help their work units or teams perform better and 9.8% disagreed. Of the health workers, 80.1% indicated that they have made suggestions to improve the overall performance of their organisations, 10.1% were undecided and 9.8% disagreed.

**Table 8:** Items for individual proactive behaviours (n = 276)

**Item description**	**Disagree**		**Undecided**		**Agree**		**Total**	
	**n**	**%**	**n**	**%**	**n**	**%**	**n**	**%**
I have initiated better ways of performing my core tasks	0	0.0	0	0.0	276	100.0	276	100.0
I have developed new and improved methods to help my work unit perform better	27	9.8	0	0.0	249	90.2	276	100.0
I have made suggestions to improve the overall performance of the organisation	27	9.8	28	10.1	221	80.1	276	100.0

The results clearly indicate that the health workers are proactive and this is likely to enhance their performance. Some studies report that workers who score highly on creative performance or are proactive achieve high outcomes in innovative effectiveness [28]. This is good for individual, team, and organisational performance. Proactive behaviours ensure that the whole organisation develops rather than only individual departments [29].

#### Client-oriented behaviours

Table 9 shows that all health workers (100.0%) are aware of their task of serving the clients, have the clients’ interest in mind and respect their clients’ opinions. All the health workers (100.0%) indicated that they always behave professionally, take a problem-solving approach to care for their clients and give their clients opportunity to express their needs. The majority of health workers (99.3%) agreed that they try to assess their clients’ needs and 90.2% have respect for their clients. Ninety percent of the health workers indicated that they always take time to perform their clinical duties and try to address their clients’ needs with the appropriate treatment available.

**Table 9:** Items concerning client-oriented behaviour (n =276)

**Item description**	**Disagree**		**Undecided**		**Agree**		**Total**	
	**n**	**%**	**n**	**%**	**n**	**%**	**n**	**%**
I try to assess what the clients’ needs are	0	0.0	2	0.7	274	99.3	276	100.0
I have the clients best interest in mind	0	0.0	0	0.0	276	100.0	276	100.0
I try to address the clients’ needs with the appropriate treatment available	0	0.0	28	10.1	248	89.9	276	100.0
I am aware that my task is to serve my clients to the best of my ability	0	0.0	0	0.0	276	100.0	276	100.0
I respect what the patients have to say	0	0.0	0	0.0	276	100.0	276	100.0
I have respect for my clients	27	9.8	0	0.0	249	90.2	276	100.0
I give clients opportunity to express their needs with me	0	0.0	0	0.0	276	100.0	276	100.0
I take a problem-solving approach to care for my clients	0	0.0	0	0.0	276	100.0	276	100.0
I always take time to perform my clinical work	28	10.1	0	0.0	248	89.9	276	100.0
I always behave in a professional manner	0	0.0	0	0.0	276	100.0	276	100.0

These results demonstrate that health workers are generally inclined to display client-oriented behaviours. Client-oriented behaviours include attributes such as empathy, assurance, responsiveness, authenticity, listening, dedication and civility which the health workers in this study seem to display [30]. The behaviours of health workers such as doctors, nurses and clinical officers are critical to both patient satisfaction and successful health outcomes.

#### Correlation between individual performance behaviours and performance of health workers

The results show that individual performance behaviours are positively related to the dimensions of performance. There is a significant and high positive correlation between responsiveness and the individual proficiency behaviours of health workers (*rs* = 0.856; *P* < 0.01). There are also significant and moderate correlations between individual client-oriented behaviours (*rs* = 0.669; *P* < 0.01), individual adaptive behaviours (*rs* = 0.623; *P* < 0.01), individual proactive behaviours (*rs* = 0.598; *P* < 0.01) and the responsiveness of health workers. Indeed there is a high likelihood that health workers who are highly proficient, client-oriented and display adaptive and proactive behaviours, will demonstrate high responsiveness towards clients. The individual workers’ behaviours that involve teamwork, innovativeness and self-advancement contribute to performance [28] (see Table 10).

**Table 10:** Correlation between the individual performance behaviours and the dimensions of performance (n = 276)

**Spearman’s*rho*rank order non-parametric correlations**		**Responsiveness**	**Availability**	**Productivity**	**Competence**
**Individual proficiency behaviours**	Correlation	0.856^**^	0.724^**^	0.235^**^	0.562^**^
	Sig (2-tailed)	0.000	0.000	0.000	0.000
	N	276	276	276	276
**Individual adaptive behaviours**	Correlation	0.623^**^	0.659^**^	0.343^**^	0.643_**_
	Sig (2-tailed)	0.000	0.000	0.000	0.000
	N	276	276	276	276
**Individual proactive behaviours**	Correlation	0.598^**^	0.759^**^	0.262^**^	0.359^**^
	Sig (2-tailed)	0.000	0.000	0.000	0.000
	N	276	276	276	276
**Individual client-oriented behaviours**	Correlation	0.669^**^	0.537^**^	0.396^**^	0.449^**^
	Sig (2-tailed)	0.000	0.000	0.000	0.000
	N	276	276	276	276

^**^Correlation is significant (Sig) at the 0.01 level (2-tailed).

The results also indicate significant and high positive correlation between the individual proactive behaviours (*rs* = 0.759; *P* < 0.01), individual proficiency behaviours (*rs* = 0.724; *P* < 0.01) and the availability of health workers. There is significant and moderate correlation between the individual adaptive behaviours (*rs* = 0.659; *P* < 0.01), individual client oriented behaviours (*rs* = 0.537; *P* < 0.01) and the availability of health workers. This demonstrates that proactive and proficient individuals are likely to be available to perform their duties. There is significant but low positive correlation between individual client-oriented behaviours, (*rs* = 0.396; *P* < 0.01), individual adaptive behaviours (*rs* = 0.343; *P* < 0.01), individual proactive behaviours (*rs* = 0.262; *P* < 0.01), individual proficiency behaviours (*rs* = 0.235; *P* < 0.01) and the productivity of health workers. Indeed while adaptive, proactive and proficiency behaviours may facilitate individual productivity; it is not always certain that this will be the case (see Table 10).

The results in Table 10 further show that there are significant and moderate correlations between the individual adaptive behaviours (*rs* = 0.643; *P* < 0.01), individual proficiency behaviours (*rs* = 0.562; *P* < 0.01) and the competence of health workers. There are also significant but low correlations between the individual client-oriented behaviours (*rs* = 0.449; *P* < 0.01), individual proactive behaviours (*rs* = 0.359; *P* < 0.01) and competence of health workers. This can be interpreted as follows: adaptive and proficient health workers are highly likely to be competent but this may not always hold since competent health workers may not always be adaptive. The workers who score highly on creative performance are likely to achieve high outcomes in creative effectiveness [28].

## Conclusion

This research uncovered that the health workers under study possessed considerable degrees of the four performance measures (responsiveness, availability, productivity and competence). This indicates that the respondents understand and are capable of performing according to their clients as well as their organisations’ expectations, which is providing quality health care. Despite this, the reality is that the health workers often fall short of the stakeholders’ expectations because of factors beyond their control. These include organisational factors such as lack of adequate supplies of drugs and equipment. Even more inhibiting is the lack of professional support reported by some respondents as this is vital to maintaining spirit of high performance even when circumstances are not so favourable, especially in the decentralised facilities.

There is a need to put in place a mechanism for timely management of the clients. By so doing, the responsiveness of health services is likely to be enhanced. In addition the local governments should put in place an efficient mechanism for attracting and retaining health workers especially for the rural areas. This can be done through provision of housing in the rural facilities and possibly by introducing a hardship allowance. Lastly, the Ministry of Health has to set clear indicators for measuring the productivity of the health workers and these should be communicated to all stakeholders in the health sector. This will likely improve accountability, which is one of the key elements of decentralisation.

By implications, all stakeholders need to work together to support the performance of health workers if desired health outcomes are to be attained. The central government needs to exercise full fiscal decentralisation to empower local governments and facilities so that they can provide health workers with adequate supplies and training.

## Abbreviations

HSSP: Health Sector Strategic Plan; IMCI: Integrated Management of Childhood Illnesses; MDGs: Millennium Development goals; Rs: Spearman rho correlation; SPSS: Statistical Package for Social Sciences; TB: Tuberculosis; UNCST: Uganda National Council for Science and Technology; VHTs: Village Health Teams; WHO: World Health Organization.

## Competing interests

The authors declare no competing interests.

## Authors’ contributions

GWL conducted the study for his Doctoral thesis; JHR and BLD were the advisors for this study. GWL obtained permission for the study, collected, analysed and interpreted the data. JHR and BLD provided the overall supervision of this study right from its inception to its conclusion. GWL wrote the first draft of the article. Both JHR and BLD critically reviewed and contributed substantially to the article and continued with the process until publication. All authors read and approved the final manuscript.
